# Supplementary figures and images for: Defective chaperone-mediated autophagy in the retinal pigment epithelium of age-related macular degeneration patients
Source: EMBO Mol Med. 2025 Oct 30;17(12):3472–95. doi: 10.1038/s44321-025-00329-w (PMC12686442; doi:10.1038/s44321-025-00329-w)

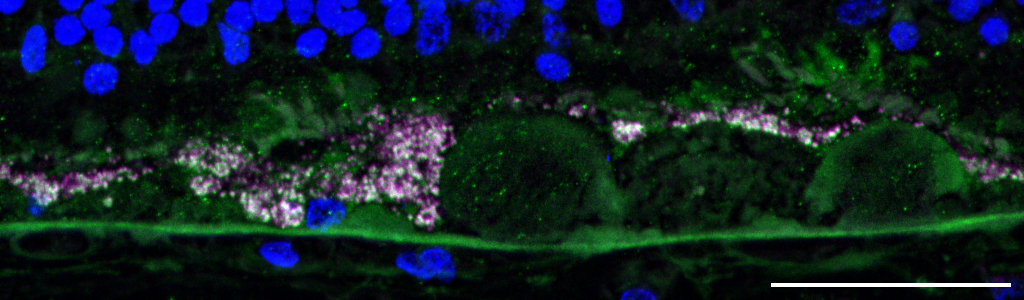

Supplement: Supplementary file 9 — Source data Fig. 1 [file 44321_2025_329_MOESM9_ESM.zip › Figure1/Figure1E/RPE_AMD_Drusen_MAX_E456_221025ODWGmacula_3-1.tif (RGB)scalebar50um.tif]

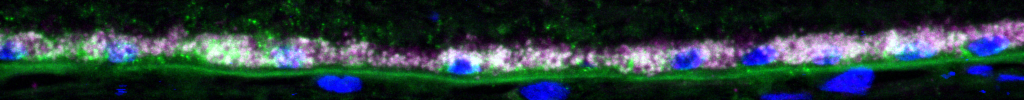

Supplement: Supplementary file 9 — Source data Fig. 1 [file 44321_2025_329_MOESM9_ESM.zip › Figure1/Figure1E/RPE_Healthy_MAX_E456_210286ODWGmacula_2-1.tif (RGB).tif]

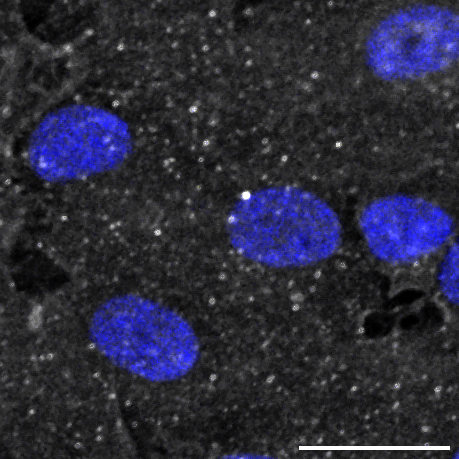

Supplement: Supplementary file 10 — Source data Fig. 2 [file 44321_2025_329_MOESM10_ESM.zip › Figure2/Figure2G/E440_ProteoStat_AMD-1scalebar25um.tif]

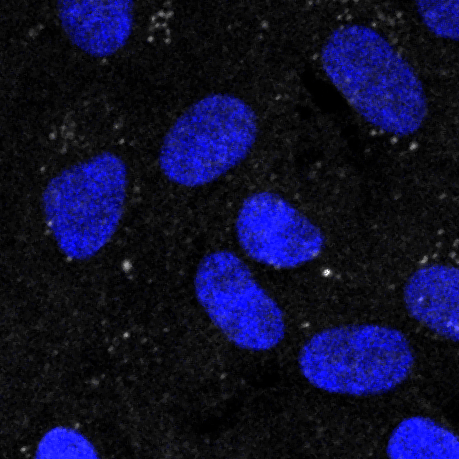

Supplement: Supplementary file 10 — Source data Fig. 2 [file 44321_2025_329_MOESM10_ESM.zip › Figure2/Figure2G/E440_ProteoStat_Healthy-1.tif]

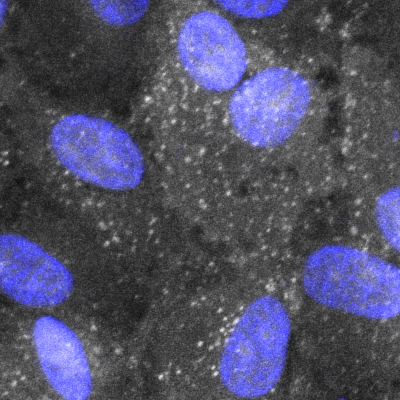

Supplement: Supplementary file 10 — Source data Fig. 2 [file 44321_2025_329_MOESM10_ESM.zip › Figure2/Figure2F/MAX_E339_S_H2_1.tif (RGB).tif]

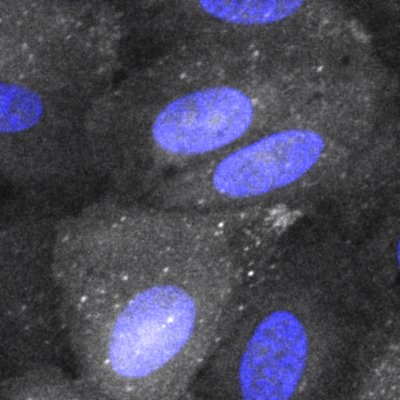

Supplement: Supplementary file 10 — Source data Fig. 2 [file 44321_2025_329_MOESM10_ESM.zip › Figure2/Figure2F/MAX_E339_H2_2-1-1.lsm (RGB).tif]

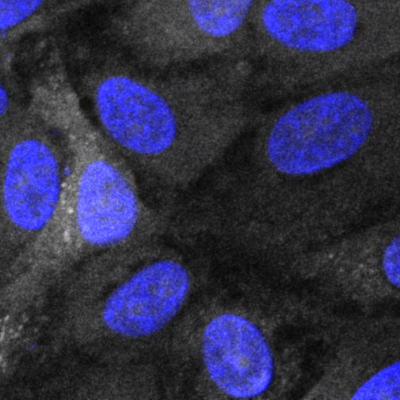

Supplement: Supplementary file 10 — Source data Fig. 2 [file 44321_2025_329_MOESM10_ESM.zip › Figure2/Figure2F/MAX_E339_A1_2-1.lsm (RGB).tif]

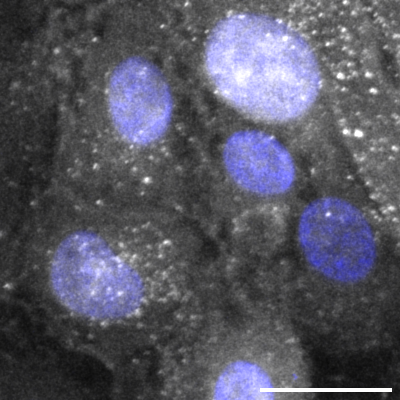

Supplement: Supplementary file 10 — Source data Fig. 2 [file 44321_2025_329_MOESM10_ESM.zip › Figure2/Figure2F/MAX_E339_S_A3_1-1.lsm (RGB)scalebar25um.tif]

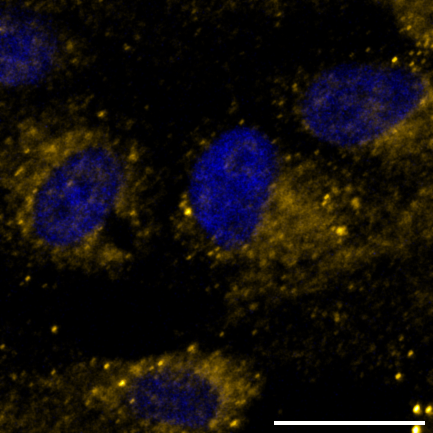

Supplement: Supplementary file 10 — Source data Fig. 2 [file 44321_2025_329_MOESM10_ESM.zip › Figure2/Figure2K/E452_4HNE_A6_1-1.tif (RGB)scalebar25um.tif]

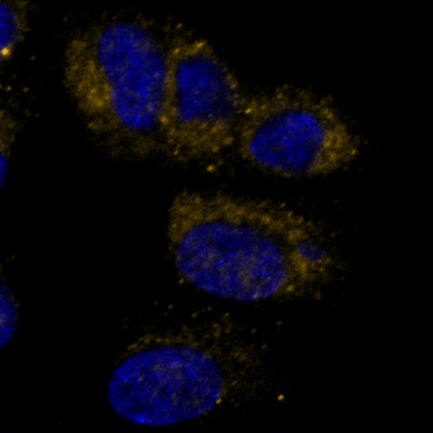

Supplement: Supplementary file 10 — Source data Fig. 2 [file 44321_2025_329_MOESM10_ESM.zip › Figure2/Figure2K/E452_4HNE_H4_1-1.tif (RGB).tif]

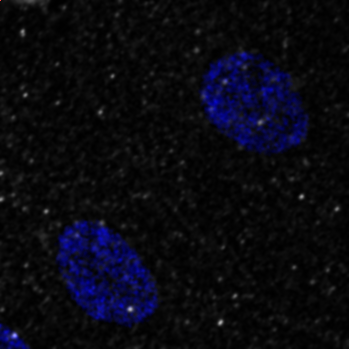

Supplement: Supplementary file 12 — Source data Fig. 4 [file 44321_2025_329_MOESM12_ESM.zip › Figure4/Figure4B/E442_H1_CA_1-1.lsm (RGB).tif]

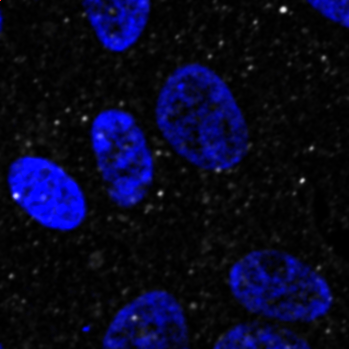

Supplement: Supplementary file 12 — Source data Fig. 4 [file 44321_2025_329_MOESM12_ESM.zip › Figure4/Figure4B/E442_H3_Veh_1-1.lsm (RGB).tif]

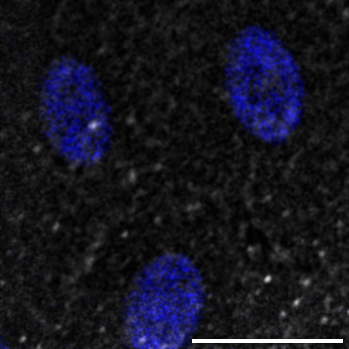

Supplement: Supplementary file 12 — Source data Fig. 4 [file 44321_2025_329_MOESM12_ESM.zip › Figure4/Figure4B/E452_ProteoStat_A4_1-1.lsm (RGB)scalebar25um.tif]

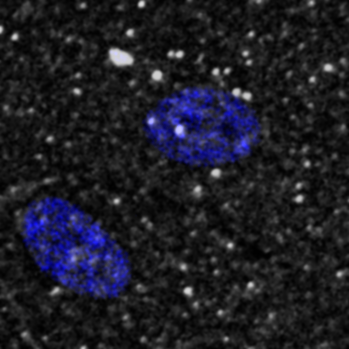

Supplement: Supplementary file 12 — Source data Fig. 4 [file 44321_2025_329_MOESM12_ESM.zip › Figure4/Figure4B/E452_ProteoStat_Veh_A4_1-1.lsm (RGB).tif]

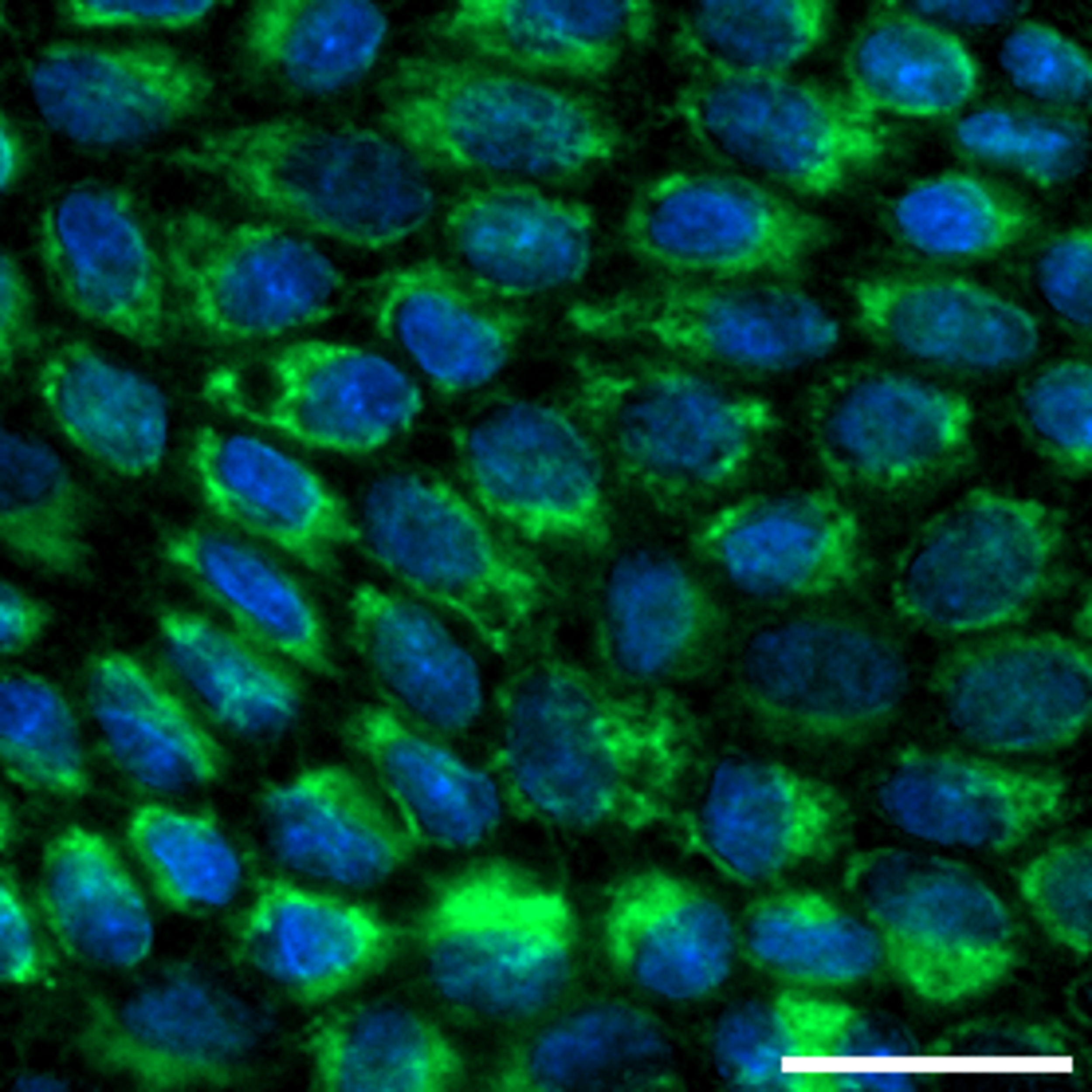

Supplement: Supplementary file 12 — Source data Fig. 4 [file 44321_2025_329_MOESM12_ESM.zip › Figure4/Figure4F/MTG_AMD_CA.tiff]

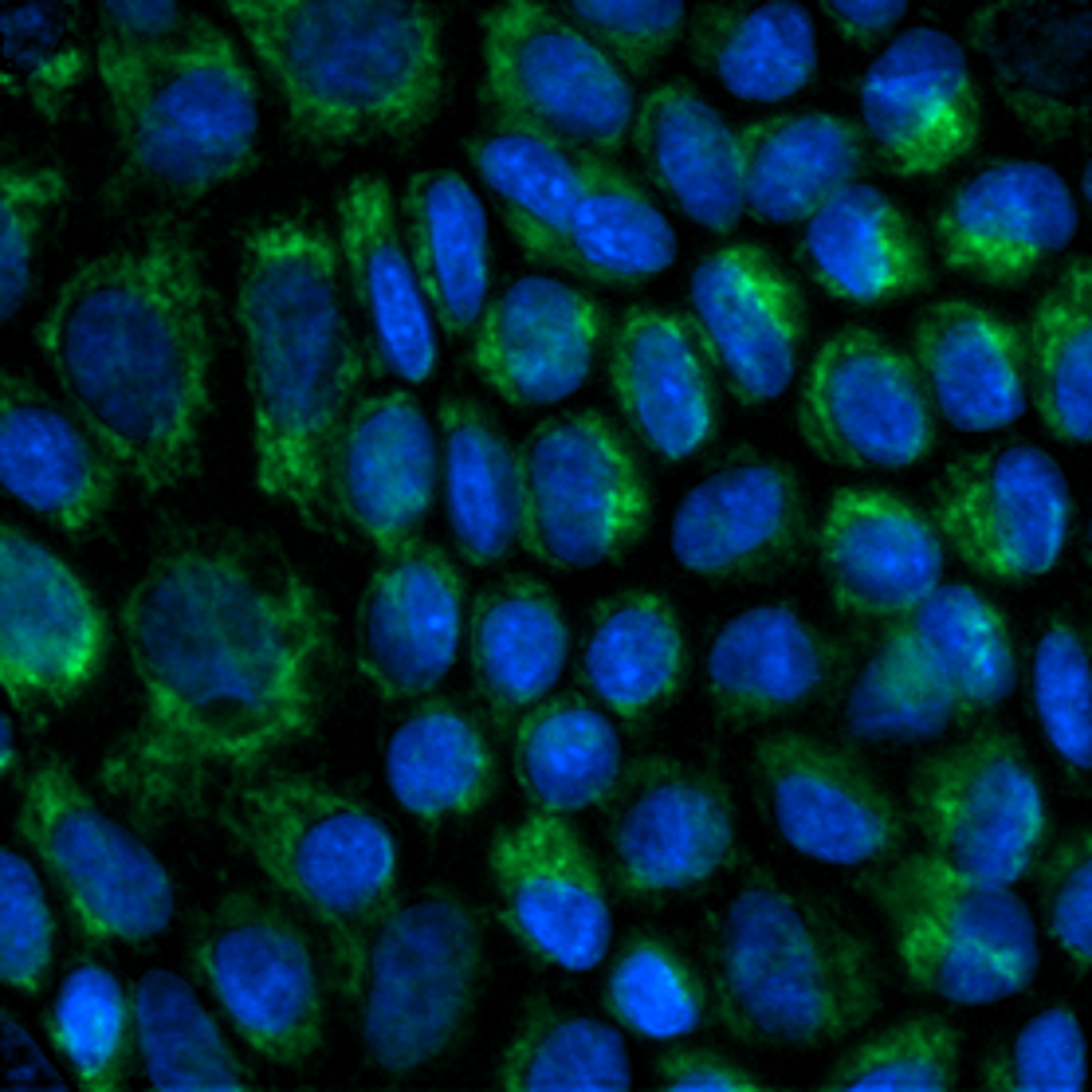

Supplement: Supplementary file 12 — Source data Fig. 4 [file 44321_2025_329_MOESM12_ESM.zip › Figure4/Figure4F/MTG_Healthy_AMD.tiff]

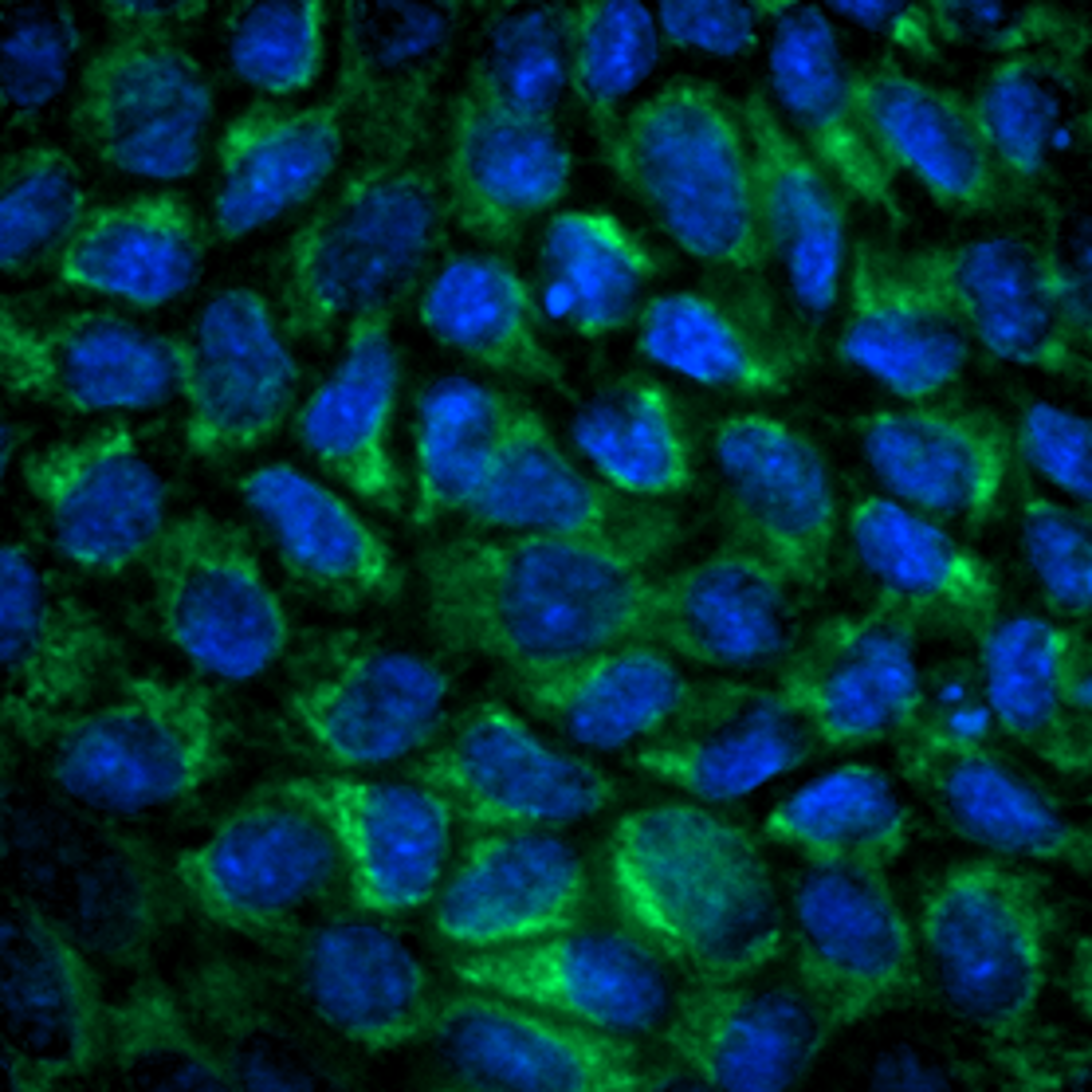

Supplement: Supplementary file 12 — Source data Fig. 4 [file 44321_2025_329_MOESM12_ESM.zip › Figure4/Figure4F/MTG_Healthy_CA.tiff]

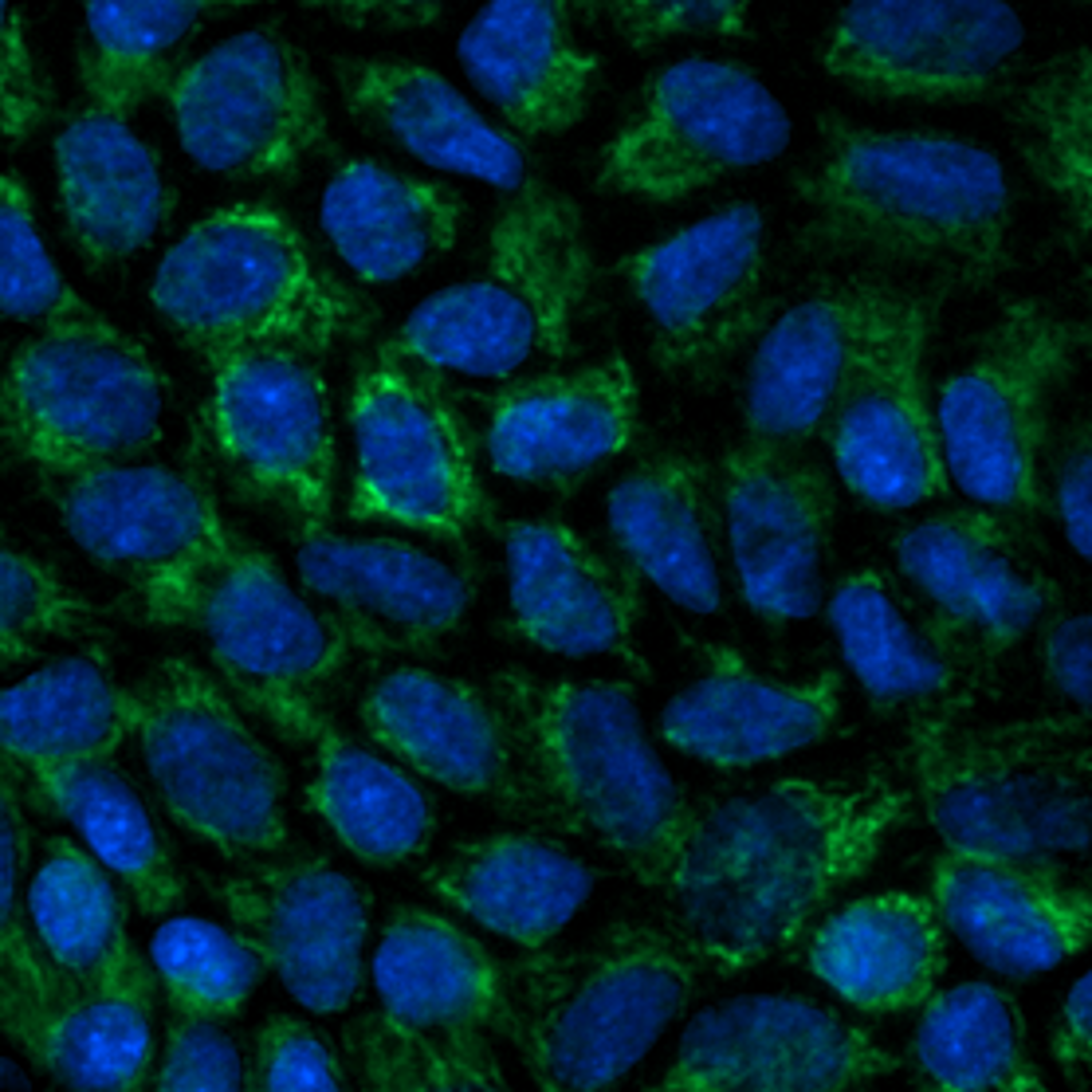

Supplement: Supplementary file 12 — Source data Fig. 4 [file 44321_2025_329_MOESM12_ESM.zip › Figure4/Figure4F/MTG_Healthy_Veh.tiff]

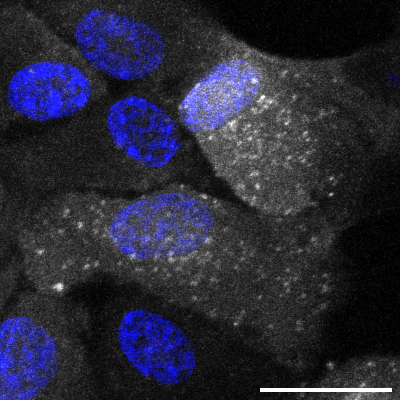

Supplement: Supplementary file 12 — Source data Fig. 4 [file 44321_2025_329_MOESM12_ESM.zip › Figure4/Figure4A/MAX_E353_CA_A7_2.lsm (RGB)scalebar25um.tif]

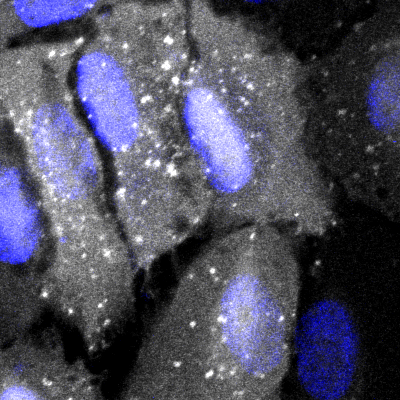

Supplement: Supplementary file 12 — Source data Fig. 4 [file 44321_2025_329_MOESM12_ESM.zip › Figure4/Figure4A/MAX_E353_CA_H5_1.lsm (RGB).tif]

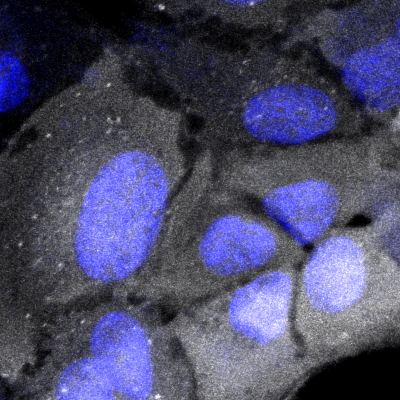

Supplement: Supplementary file 12 — Source data Fig. 4 [file 44321_2025_329_MOESM12_ESM.zip › Figure4/Figure4A/MAX_E353_Veh_A5_2.lsm (RGB).tif]

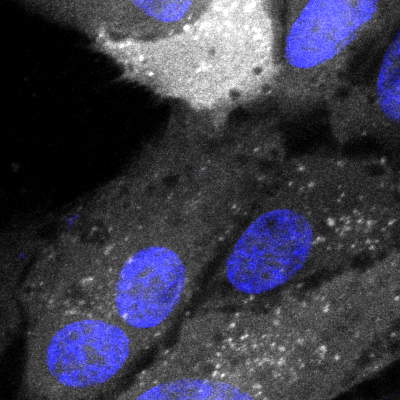

Supplement: Supplementary file 12 — Source data Fig. 4 [file 44321_2025_329_MOESM12_ESM.zip › Figure4/Figure4A/MAX_E353_Veh_H5_2.lsm (RGB).tif]

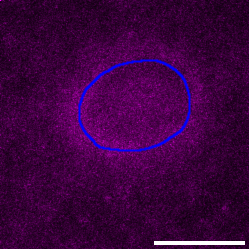

Supplement: Supplementary file 13 — Source data Fig. 5 [file 44321_2025_329_MOESM13_ESM.zip › Figure5/Figure5B/MAX_E458_NRF2_CA_A7_1-1-2_scalebar15um.tif]

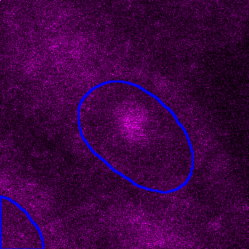

Supplement: Supplementary file 13 — Source data Fig. 5 [file 44321_2025_329_MOESM13_ESM.zip › Figure5/Figure5B/MAX_E458_NRF2_CA_H4_1-1-2.tif]

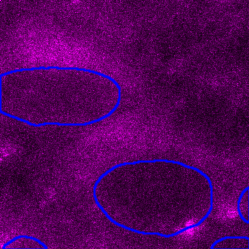

Supplement: Supplementary file 13 — Source data Fig. 5 [file 44321_2025_329_MOESM13_ESM.zip › Figure5/Figure5B/MAX_E458_NRF2_Veh_A5_1-2.tif]

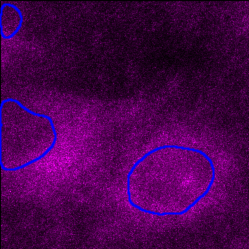

Supplement: Supplementary file 13 — Source data Fig. 5 [file 44321_2025_329_MOESM13_ESM.zip › Figure5/Figure5B/MAX_E458_NRF2_Veh_H5_1-1-2.tif]

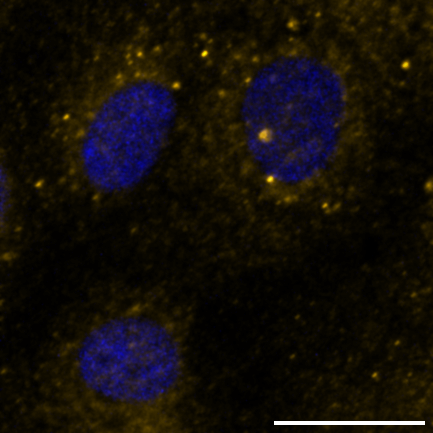

Supplement: Supplementary file 13 — Source data Fig. 5 [file 44321_2025_329_MOESM13_ESM.zip › Figure5/Figure5D/E452_CA_4HNE_A7_1-1.lsm (RGB)scalebar25um.tif]

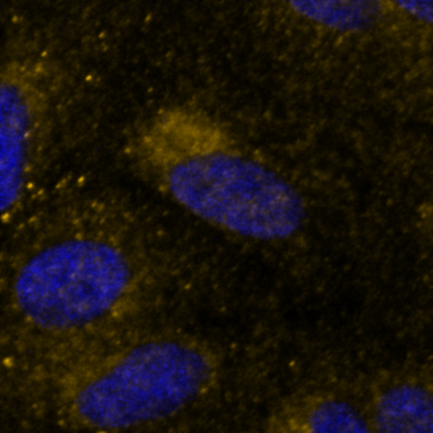

Supplement: Supplementary file 13 — Source data Fig. 5 [file 44321_2025_329_MOESM13_ESM.zip › Figure5/Figure5D/E452_CA_4HNE_H5_2-1.lsm (RGB).tif]

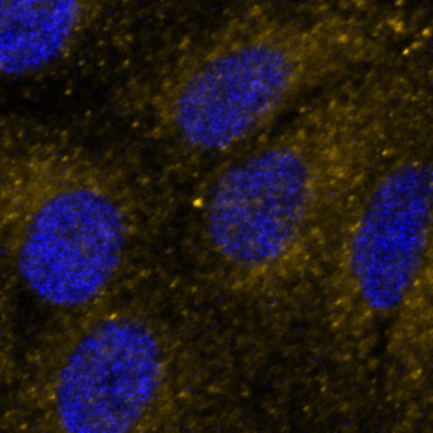

Supplement: Supplementary file 13 — Source data Fig. 5 [file 44321_2025_329_MOESM13_ESM.zip › Figure5/Figure5D/E452_Veh_4HNE_A5_1-1.lsm (RGB).tif]

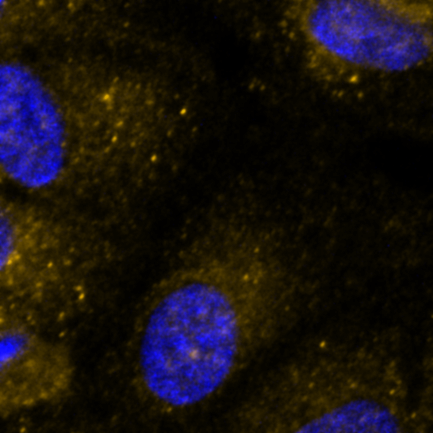

Supplement: Supplementary file 13 — Source data Fig. 5 [file 44321_2025_329_MOESM13_ESM.zip › Figure5/Figure5D/E452_Veh_4HNE_H5_1-1.lsm (RGB).tif]

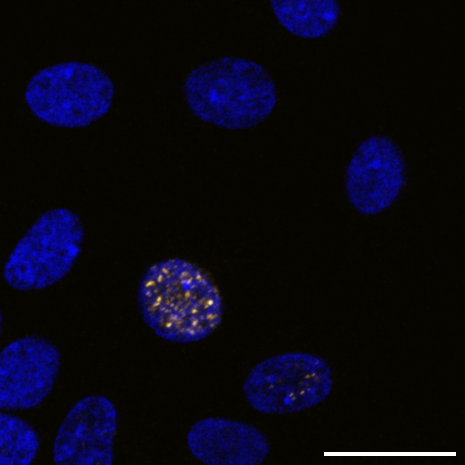

Supplement: Supplementary file 13 — Source data Fig. 5 [file 44321_2025_329_MOESM13_ESM.zip › Figure5/Figure5F/MAX_E458_CA_A4_2-1.tif (RGB)scalebar25um.tif]

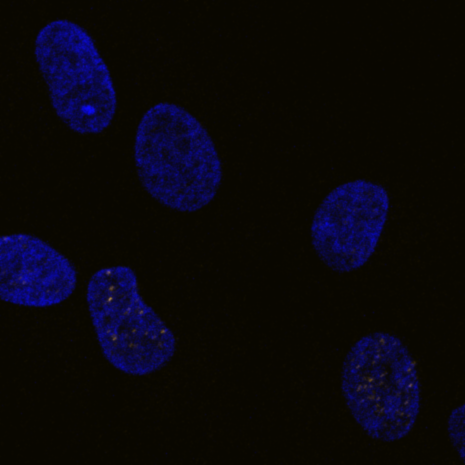

Supplement: Supplementary file 13 — Source data Fig. 5 [file 44321_2025_329_MOESM13_ESM.zip › Figure5/Figure5F/MAX_E458_CA_H5_1-1.tif (RGB).tif]

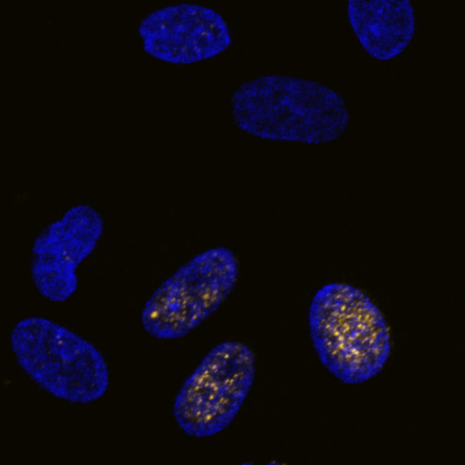

Supplement: Supplementary file 13 — Source data Fig. 5 [file 44321_2025_329_MOESM13_ESM.zip › Figure5/Figure5F/MAX_E458_Veh_A6_1-1.tif (RGB).tif]

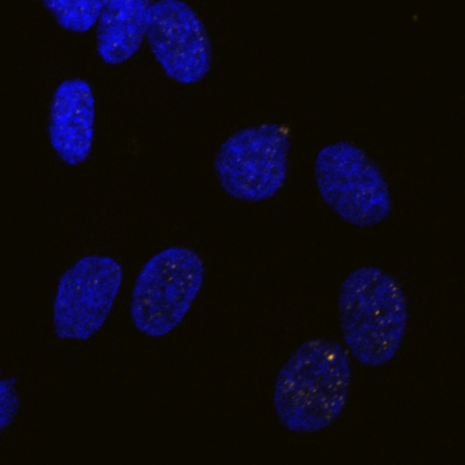

Supplement: Supplementary file 13 — Source data Fig. 5 [file 44321_2025_329_MOESM13_ESM.zip › Figure5/Figure5F/MAX_E458_Veh_H5_1-1.tif (RGB).tif]
