# Supplementary material for: Defective chaperone-mediated autophagy in the retinal pigment epithelium of age-related macular degeneration patients
Source: EMBO Mol Med. 2025 Oct 30;17(12):3472–95. doi: 10.1038/s44321-025-00329-w (PMC12686442; doi:10.1038/s44321-025-00329-w)
Supplement: Supplementary file 19 — Expanded View Figures [file 44321_2025_329_MOESM19_ESM.pdf]

Expanded View Figures

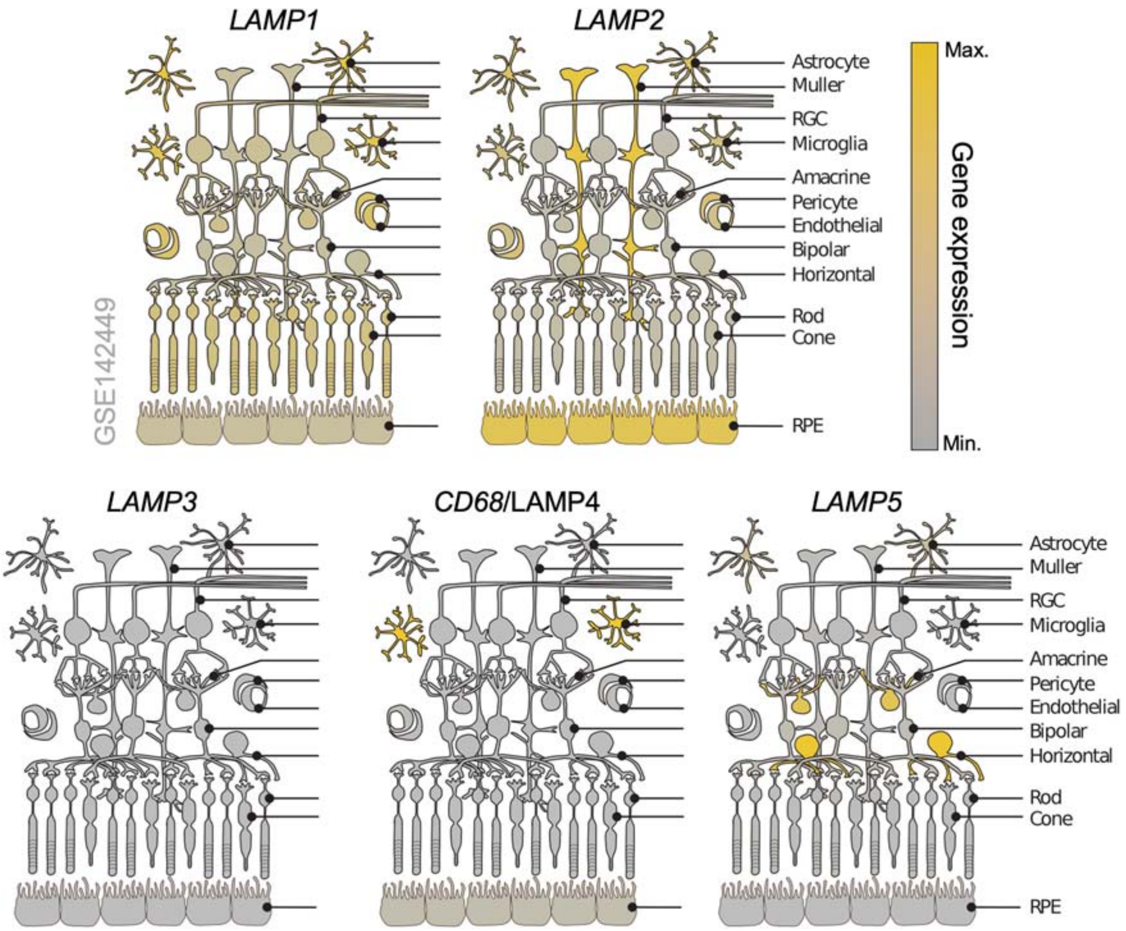

Figure EV1. *LAMP2* is preferentially expressed in the RPE and glial cells.

Visual heatmap of mRNA levels of different LAMPs in the neuroretina and RPE, generated using *Spectacle* and publicly available scRNA-seq data (GSE142449).

 © The Author(s)

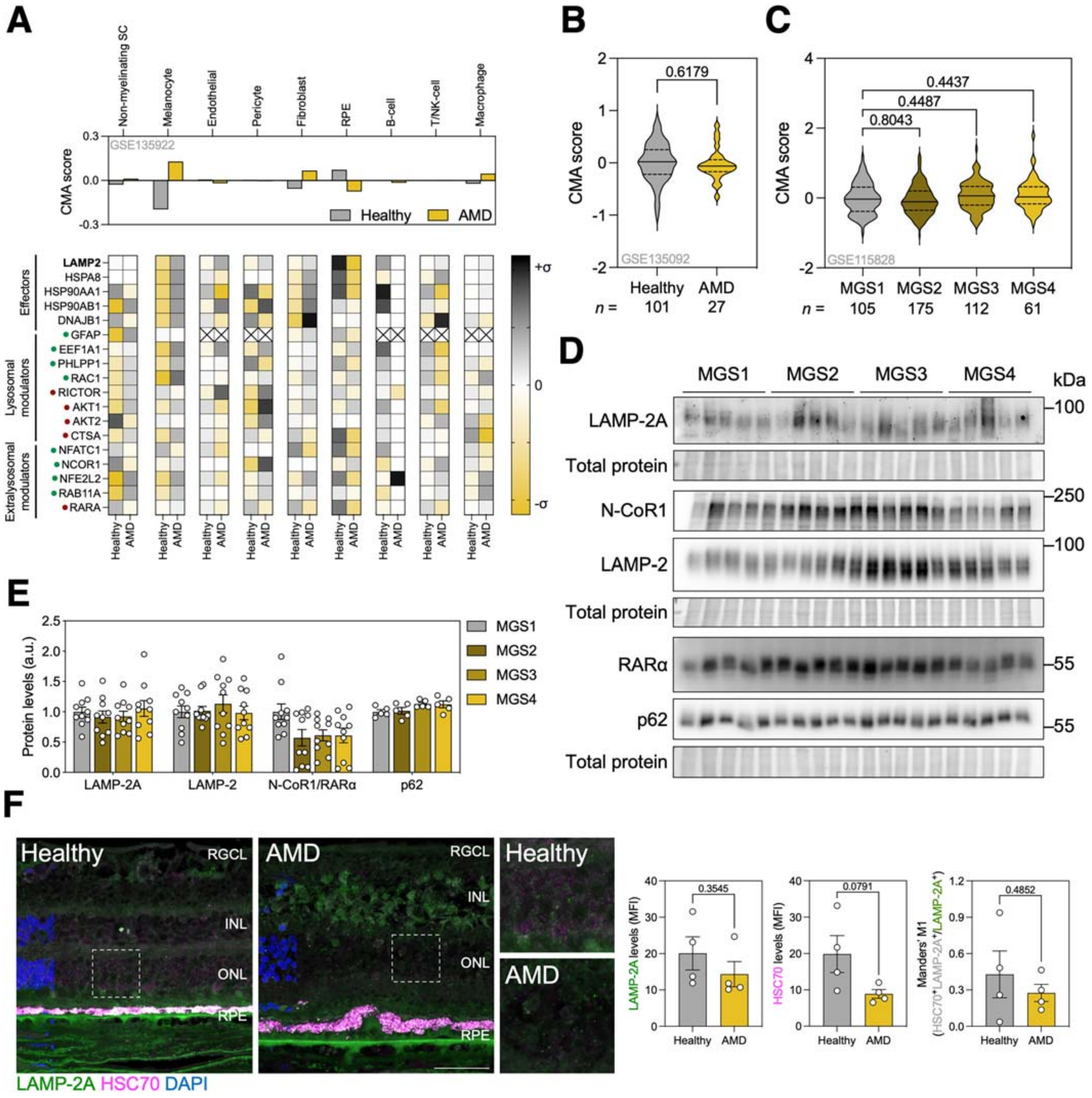

Figure EV2. CMA is not affected in other cell types within the RPE/choroid interface or the neuroretina.

(**A**) Heatmap showing the mRNA levels of the CMA network components (bottom) and CMA activation score obtained from a publicly available RNA-seq dataset (GSE135922; top). (**B**) CMA activation score in the macular neuroretina obtained from a publicly available RNA-seq dataset (GSE135092). (**C**) CMA activation score in the neuroretina of MGS-graded donors obtained from a publicly available scRNA-seq dataset (GSE115828). (**D**) Western blot analysis of CMA-related proteins (LAMP-2A, N-CoR1, RARα, LAMP-2) and macroautophagic substrates (p62) in the neuroretina of MGS-graded donors. (**E**) Quantification as shown in (**D**). ($n = 5$–10). (**F**) Representative images and quantification of donor sections immunostained against LAMP-2A (green) and HSC70 (magenta), nuclei were counterstained with DAPI (blue). Quantification of the levels of both proteins (MFI) and the proportion of lysHSC70 CMA-proficient lysosomes in the neuroretina is shown. ($n = 4$). Scale bar, 50 µm. All data are expressed as the mean ± s.e.m. Dots represent individual donors. p values were calculated using unpaired Student's t test ((**B**), **F** (LAMP-2A, HSC70)), Kruskal–Wallis with Dunn's post hoc test (**C**) or Mann–Whitney's U-test ((**F**) (Manders' M1)). Source data are available online for this figure.

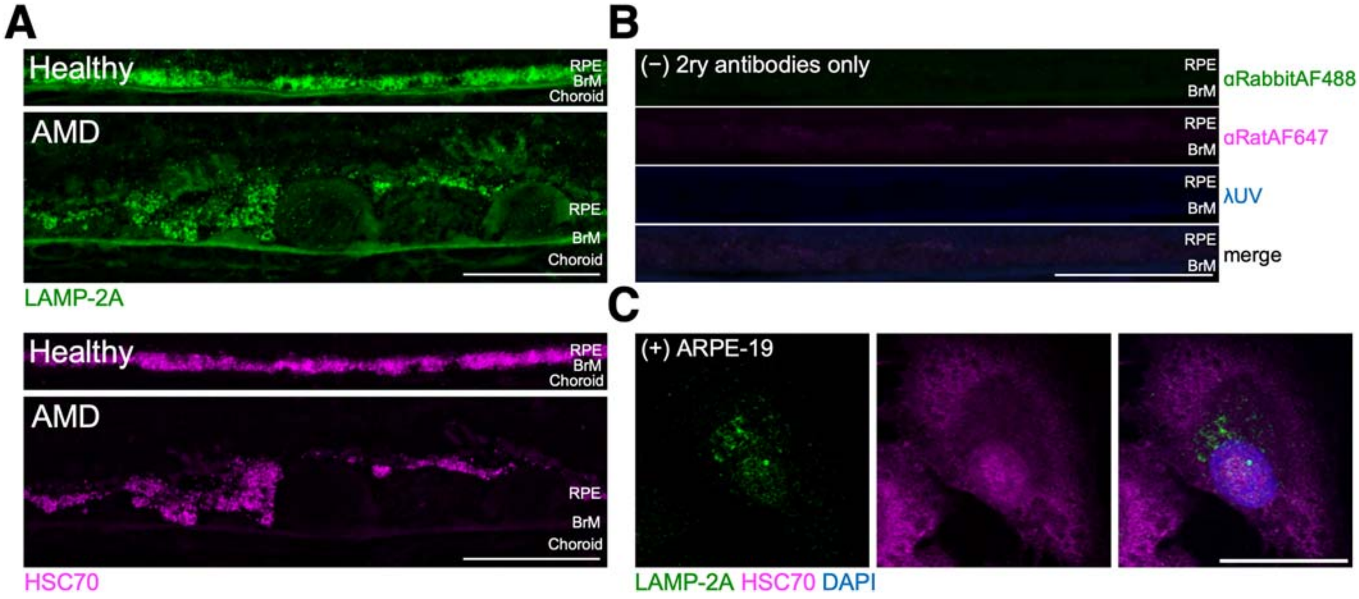

Figure EV3. LAMP-2A and HSC70 immunofluorescence in the RPE of healthy and AMD donors.

(**A**) Single-channel images of LAMP-2A (top, green) and HSC70 (bottom, magenta) immunostaining of donor eyes as shown in Fig. 1E. (**B**) Negative secondary antibody-only control. (**C**) ARPE-19 human cells immunostained against LAMP-2A (green) and HSC70 (magenta), nuclei were counterstained with DAPI (blue). Scale bars, 50 (**A**, **B**) and 25 (**C**) µm.

 © The Author(s)

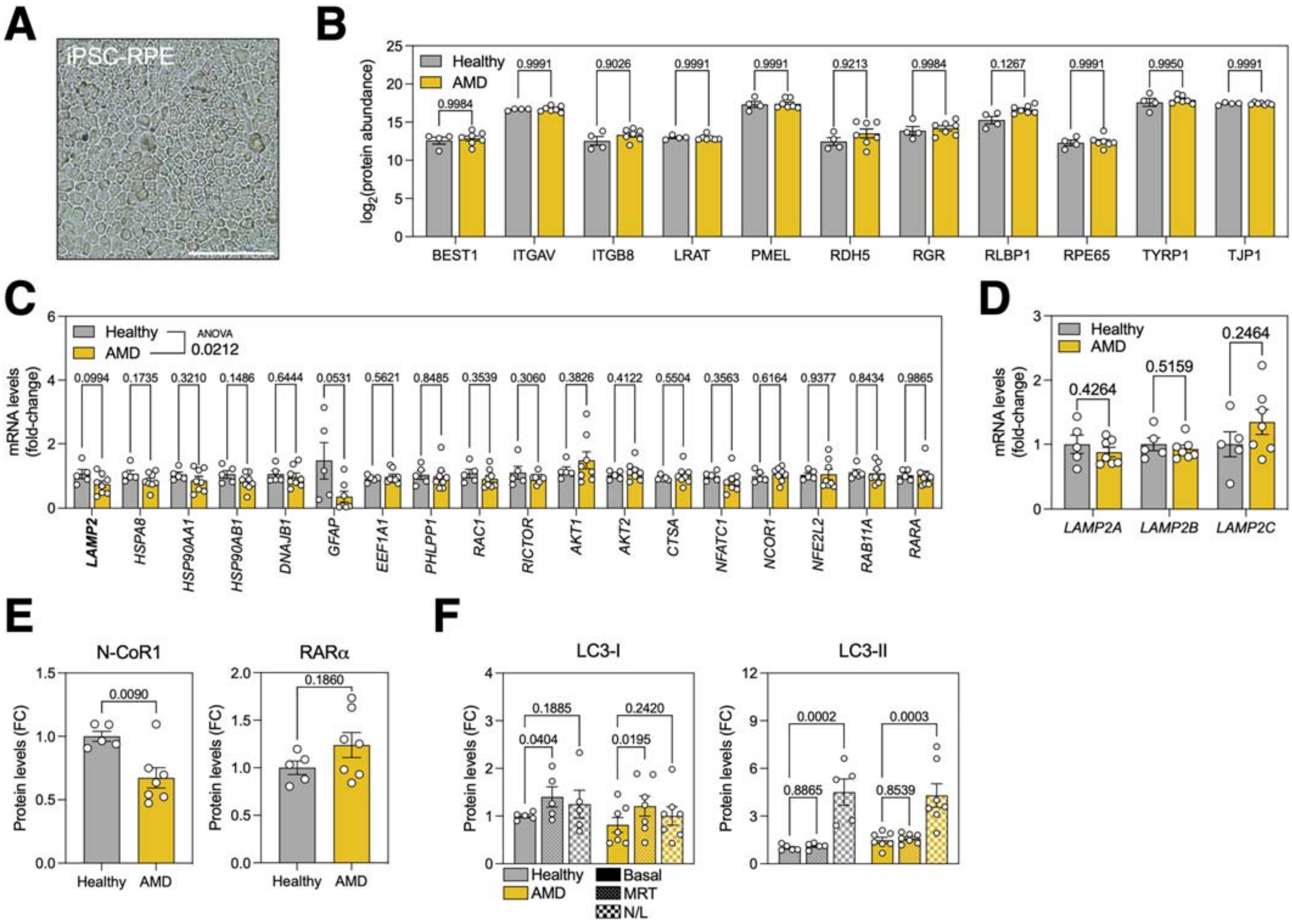

Figure EV4. Expression levels of CMA network components in iPSC-RPE.

(**A**) Representative bright field image of differentiated iPSC-RPE at P3. (**B**) Validation of RPE identity by measuring the levels of prototypic RPE proteins by bulk proteomics. ($n = 4$–7). (**C**) mRNA levels of CMA network components used for CMA activation score calculation in iPSC-RPE, as shown in Fig. 2B. ($n = 5$–8). (**D**) mRNA levels of the different LAMP2 splicing variants in iPSC-RPE. ($n = 5$–7). (**E**) Quantification of protein levels of N-CoR1 and RARα, as shown in Fig. 2C. ($n = 5$–7). (**F**) Quantification of protein levels of LC3-I and LC3-II, as shown in Fig. 3E. ($n = 5$–7). Scale bar, 100 µm. All data are expressed as the mean ± s.e.m. Dots represent individual donors. *p* values were calculated using unpaired Student's *t* test (**B**–**E**) or two-way ANOVA (**F**). Source data are available online for this figure.

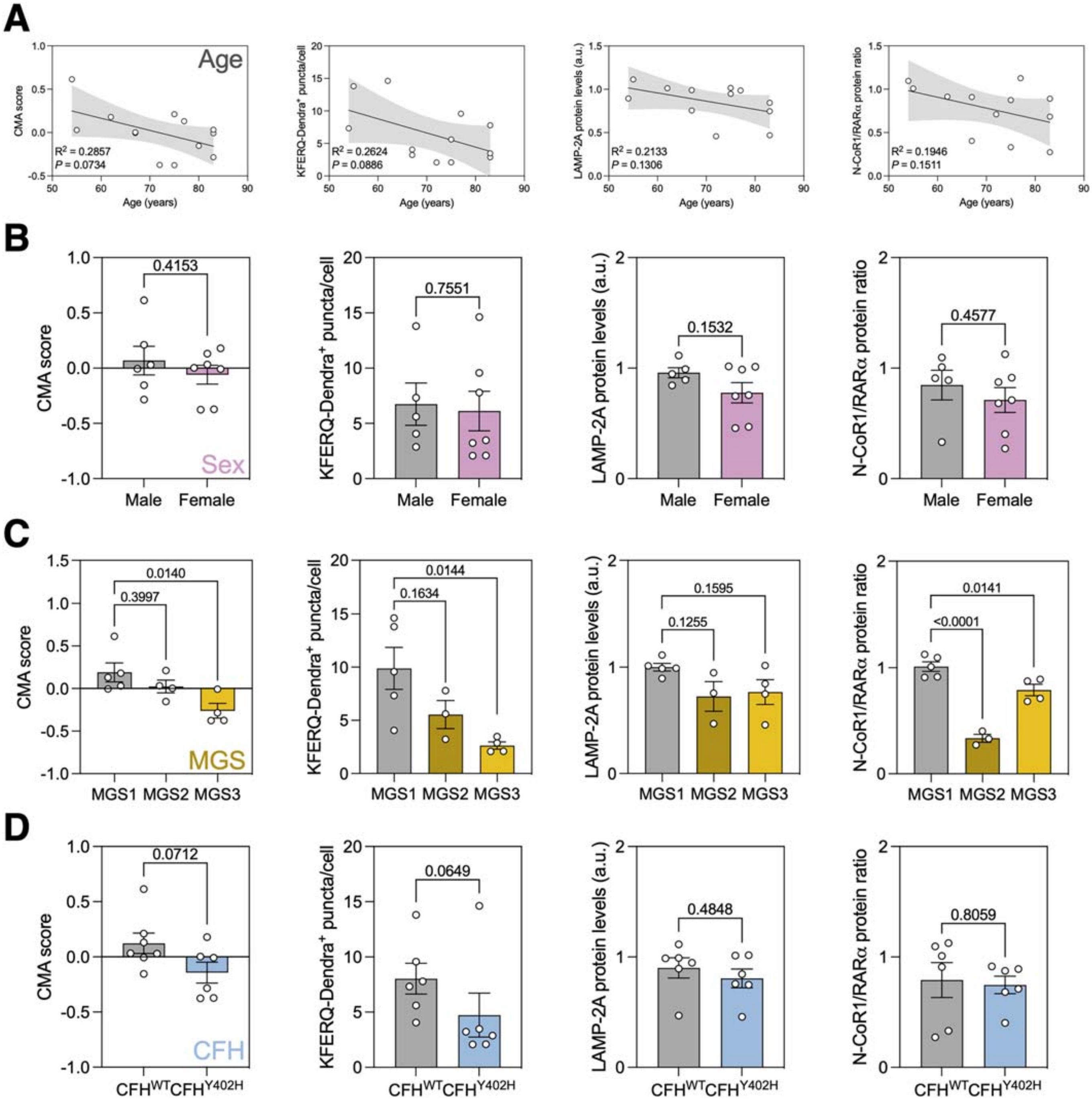

Figure EV5. CMA activity in the RPE is impacted by age and AMD severity.

Analysis of CMA activation, KFERQ-Dendra+ puncta/cell, LAMP-2A protein levels and N-CoR1/RARα ratio in iPSC-RPE classified according to donor's age (**A**), sex (**B**), MGS grading (**C**), and presence of the high-risk CFH^{Y402H} variant (**D**). ($n = 4$–12). All data are expressed as the mean ± s.e.m. Dots represent individual donors. *p* values were calculated using simple linear regression (**A**), unpaired Student's *t* test (**B** [CMA score, LAMP-2A, N-CoR1/RARα], **D** [CMA score, N-CoR1/RARα]), Mann–Whitney's *U*-test (**B** [KFERQ-Dendra] **D** [KFERQ-Dendra, LAMP-2A]), or one-way ANOVA with Dunnett's post hoc test (**C**). Source data are available online for this figure.

 © The Author(s)

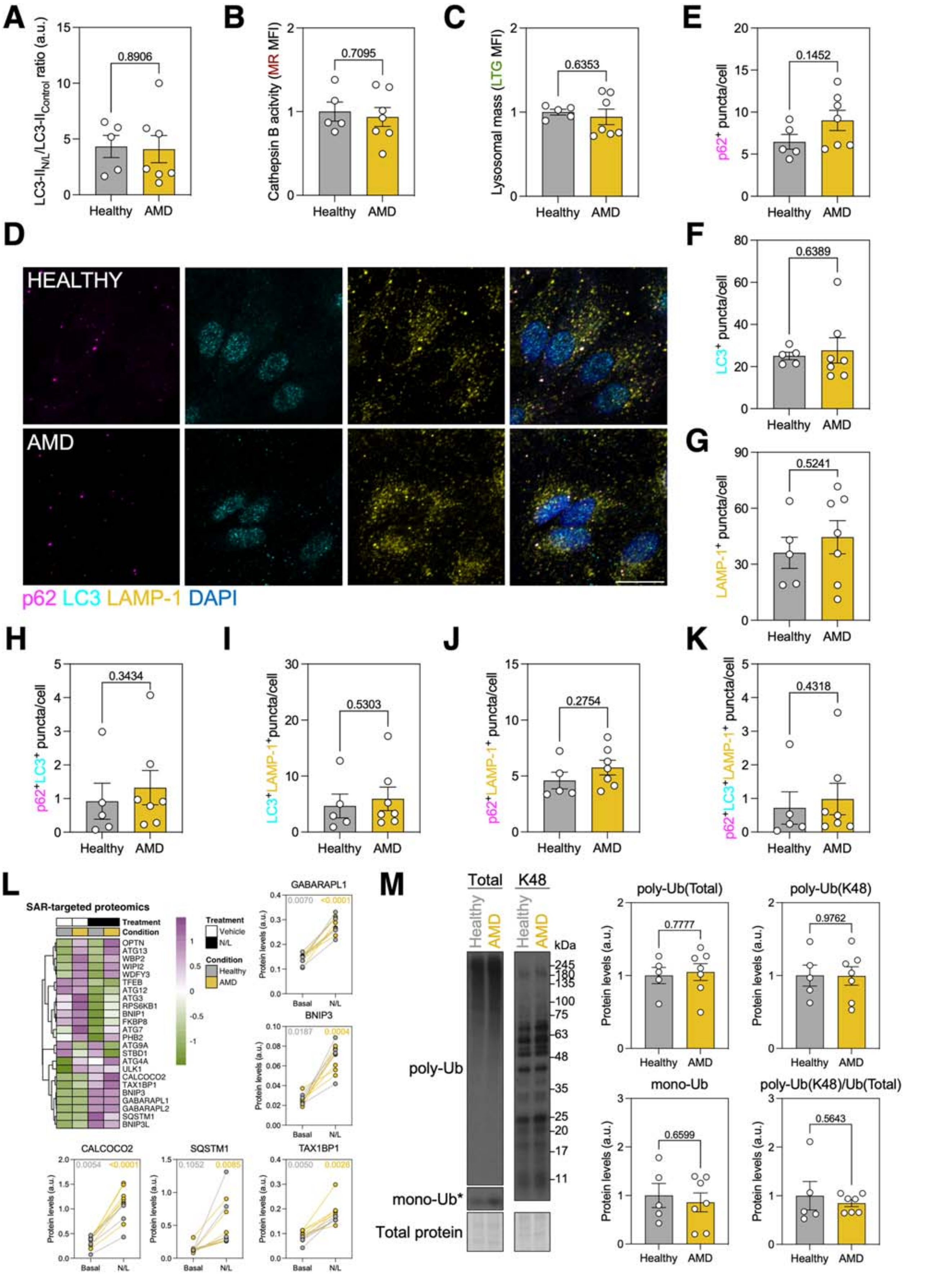
A
LC3-II N/L/LC3-II Control ratio (a.u.)
0.8906
Healthy AMD
B
Cathepsin B acitivity (MR MFI)
0.7095
Healthy AMD
C
Lysosomal mass (LTG MFI)
0.6353
Healthy AMD
E
p62+ puncta/cell
0.1452
Healthy AMD
D
HEALTHY
AMD
p62 LC3 LAMP-1 DAPI
F
LC3+ puncta/cell
0.6389
Healthy AMD
G
LAMP-1+ puncta/cell
0.5241
Healthy AMD
H
p62+LC3+ puncta/cell
0.3434
Healthy AMD
I
LC3+LAMP-1+puncta/cell
0.5303
Healthy AMD
J
p62+LAMP-1+ puncta/cell
0.2754
Healthy AMD
K
p62+LC3+LAMP-1+ puncta/cell
0.4318
Healthy AMD
L
SAR-targeted proteomics
Treatment
Condition
OPTN
ATG13
WBP2
WIPI2
WDFY3
TFEB
ATG12
ATG3
RPS6KB1
BNIP1
FKBP8
ATG7
PHB2
ATG9A
STBD1
ATG4A
ULK1
CALCOCO2
TAX1BP1
BNIP3
GABARAPL1
GABARAPL2
SQSTM1
BNIP3L
Treatment
Vehicle
N/L
Condition
Healthy
AMD
GABARAPL1
0.0070 <0.0001
BNIP3
0.0187 0.0004
CALCOCO2
0.0054 <0.0001
SQSTM1
0.1052 0.0085
TAX1BP1
0.0050 0.0026
Protein levels (a.u.)
Basal N/L
M
Total K48
Healthy AMD Healthy AMD
kDa
245
180
135
100
75
63
48
35
25
20
17
11
poly-Ub
mono-Ub*
Total protein
poly-Ub(Total)
0.7777
poly-Ub(K48)
0.9762
mono-Ub
0.6599
poly-Ub(K48)/Ub(Total)
0.5643
Protein levels (a.u.)
Healthy AMD

Figure EV6. Macroautophagy is functional in both healthy and AMD iPSC-RPE.

(**A**) Quantification of autophagic flux ($LC3\text{-}II_{N/L}/LC3\text{-}II_{Basal}$) as shown in Fig. 3E. ($n = 5–7$). (**B**) Quantification of cathepsin B proteolytic activity using Magic Red fluorogenic substrate, analyzed by live imaging. ($n = 5–7$). (**C**) Quantification of acidic lysosomal mass using LysoTracker Green, analyzed by live imaging. ($n = 5–7$). (**D**) Immunostaining analysis of $p62^+$ (magenta; macroautophagic cargo), $LC3^+$ (cyan; autophagosomes) and LAMP-1^+ (yellow; lysosomes) vesicles in iPSC-RPE, nuclei were counterstained with DAPI (blue). (**E–K**) Quantification of the number of single-, double-, and triple-positive puncta per cell as shown in **D**. ($n = 5–7$). (**L**) Heatmap and representative graphs of selective autophagy receptor (SAR) targeted proteomics of iPSC-RPE treated for 24 h with N/L. ($n = 4–7$). (**M**) Western blot analysis of the levels of total mono-ubiquitin, total poly-ubiquitinated (left), and K48-linked poly-ubiquitinated (right) proteins. ($n = 5–7$). Scale bar, 25 µm. All data are expressed as the mean ± s.e.m. Dots represent individual donors. *p* values were calculated using unpaired Student's *t* test (**A–C**, **E**, **G**, **J**, **M**), Mann–Whitney's *U*-test (**F**, **H**, **I**, **K**), or paired Student's *t* test (**L**). Source data are available online for this figure.

 © The Author(s)

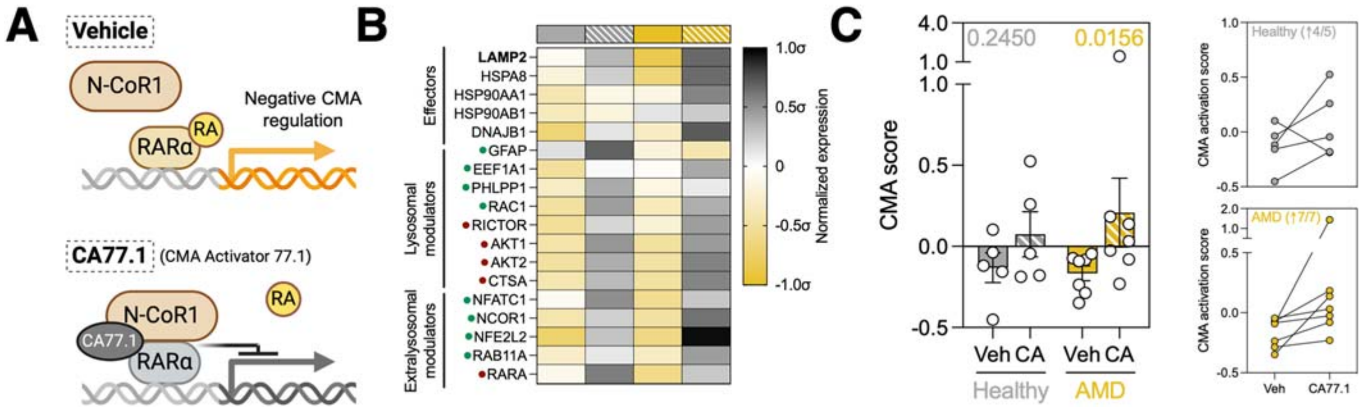

Figure EV7. CMA activator CA77.1 induces CMA in iPSC-RPE.

(**A**) Diagram depicting the mechanism of action of CA77.1, which, by stabilizing the interaction between N-CoR1 and RARα, downregulates a subset of transcriptional retinoic acid (RA) signaling involved in CMA inhibition. (**B**) Heatmap showing the mRNA levels of the CMA network components in iPSC-RPE treated with 10 μM CA77.1 for 24 h. (**C**) CMA activation score in iPSC-RPE treated with CA77.1. ($n = 5$–7). All data are expressed as the mean ± s.e.m. Dots represent individual donors. *p* values were calculated using paired Student's *t* test. Source data are available online for this figure.

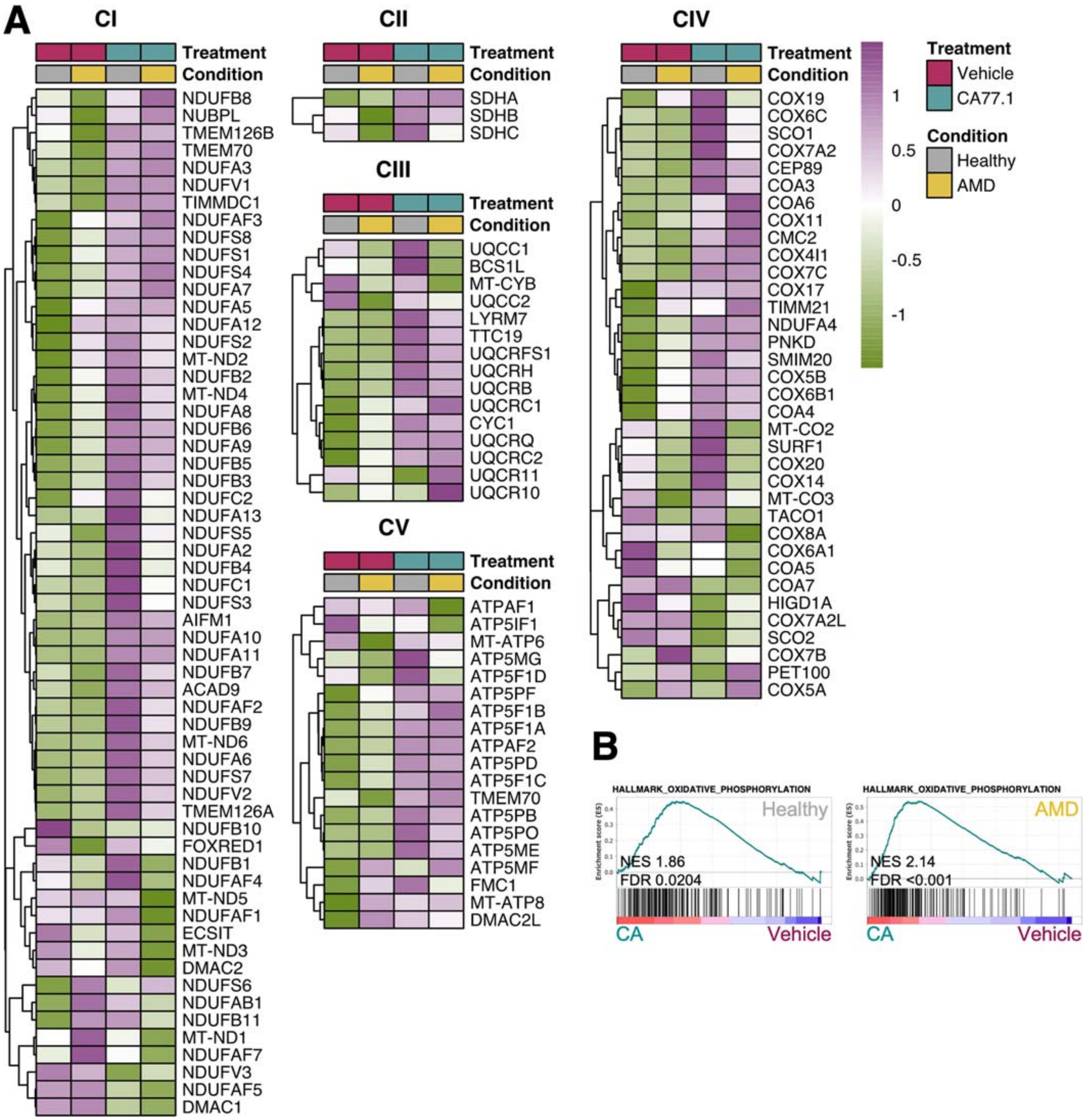

Figure EV8. CA77.1 induces mitochondrial biogenesis.

(**A**) Heatmaps showing the protein levels of all detected components of mitochondrial complexes (CI–V) of the ETC in iPSC-RPE treated with 10 μM CA77.1 for 24 h. (**B**) Upregulation of Hallmark Oxidative phosphorylation pathway in healthy and AMD iPSC-RPE analyzed by GSEA.

 © The Author(s)
